# Endoscopic management of tracheoesophageal prosthesis-induced esophageal mucosal bridge

**DOI:** 10.1055/a-2055-9768

**Published:** 2023-04-11

**Authors:** Mahmoud Y. Madi, Matthew Peller, Michael Presti, Ahmad Najdat Bazarbashi

**Affiliations:** 1 Gastroenterology and Hepatology, Saint Louis University School of Medicine, St. Louis, Missouri, United States; 2 Division of Gastroenterology and Hepatology, Washington University School of Medicine, St. Louis, Missouri, United States; 3 Gastroenterology and Hepatology, John Cochran Veteranʼs Administration Medical Center, St. Louis, Missouri, United States

Esophageal mucosal bridge (EMB) is a rare, often incidental finding encountered during esophagogastroduodenoscopy (EGD). It can be of congenital origin, or occur secondarily to local esophageal trauma, radiation therapy, and various inflammatory conditions involving the esophageal mucosa 1 . While mostly asymptomatic, EMB can often result in dysphagia by causing luminal obstruction. We present a case of symptomatic EMB secondary to long-standing tracheoesophageal voice prosthesis (TEVP) that was successfully treated with endoscopic resection using a scissor-type dissection knife.

A 77-year-old man with a history of recurrent squamous cell carcinoma of the vocal cords, which required laryngectomy, left pectoralis flap, tracheoesophageal puncture for TEVP, and chemoradiation, presented with progressive dysphagia to solid foods. Ear, nose, and throat evaluation confirmed EMB, which was dilated with rigid dilator to 16.5 mm without symptomatic relief. EGD revealed a complete EMB that was 2 cm in thickness at 17 cm from the incisors ( Fig. 1 ). The endoscope was able to pass on either side of the bridge. Immediately adjacent to the bridge, a small fistulous opening, consistent with TEVP fistula site, was noted ( Fig. 2 ). The esophagus was normal distal to this area. The decision was to proceed with dissection of the mucosal bridge.

**Fig. 1:**
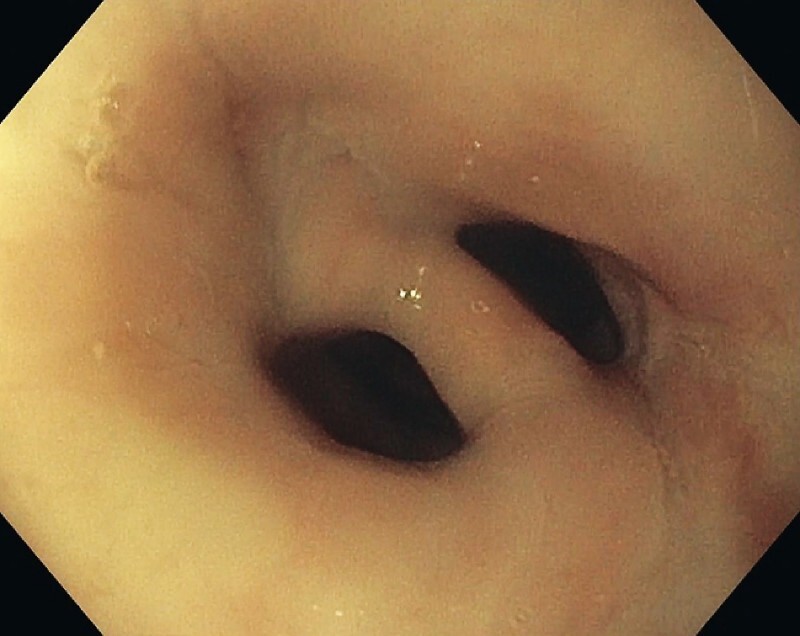
Endoscopic finding of complete esophageal mucosal bridge.

**Fig. 2:**
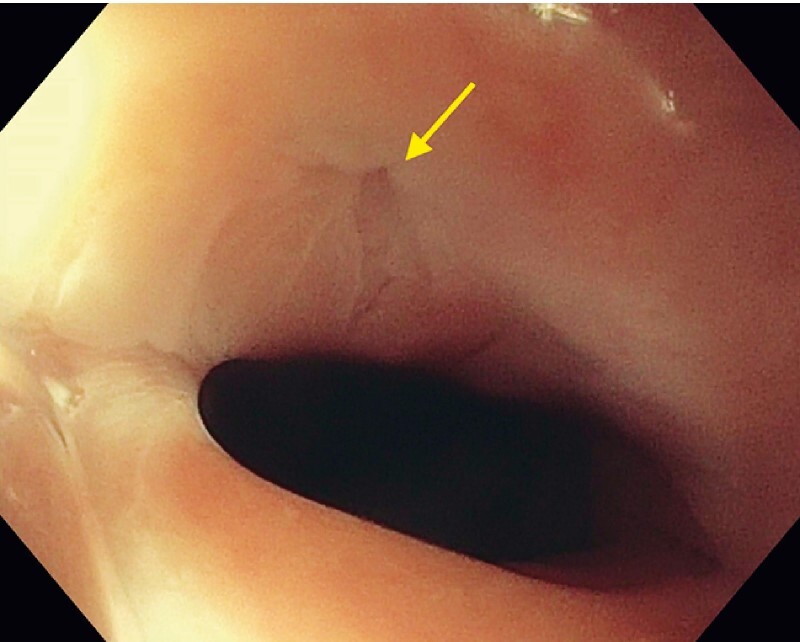
Tracheoesophageal voice prosthesis fistula site (arrow).

The bridge was injected with epinephrine with adequate blanching, followed by dissection using a scissor-type through-the-scope dissection knife (SB-Knife; Olympus, Center Valley, Pennsylvania, USA) using Endocut settings ( Fig. 3 ). This was done in a similar fashion to a Zenker’s septotomy 2 . This resulted in successful complete disruption of the bridge with no bleeding or evidence of mucosal or muscle injury ( Video 1 ).

**Fig. 3:**
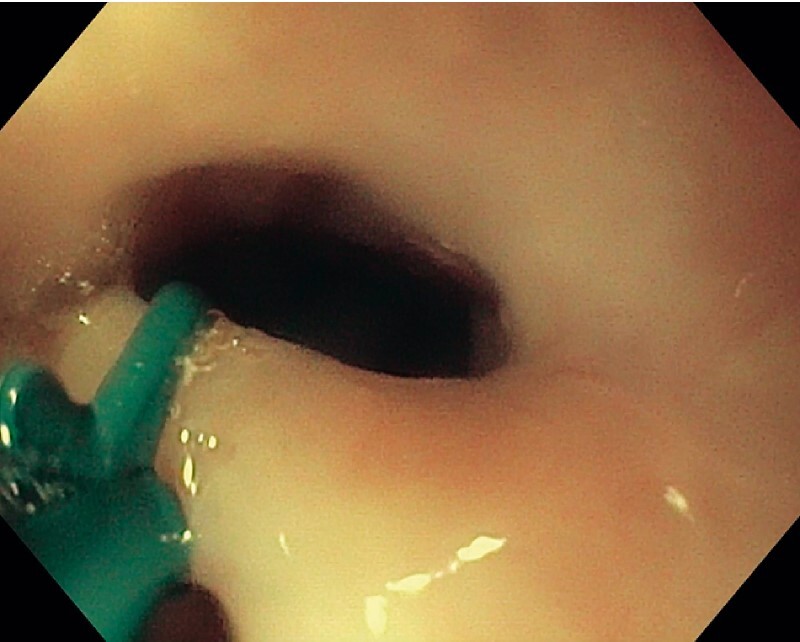
Through-the-scope dissection knife (SB-Knife; Olympus, Center Valley, Pennsylvania, USA).

**Video 1** Endoscopic examination of esophageal mucosal bridge followed by epinephrine injection and dissection with scissor-type electrocautery knife, resulting in complete disruption of the bridge without recurrence on follow-up endoscopy.


The patient reported significant improvement in dysphagia. Repeat EGD at 6 weeks revealed complete disruption of the EMB with absence of bridge regrowth ( Fig. 4 ).

**Fig. 4:**
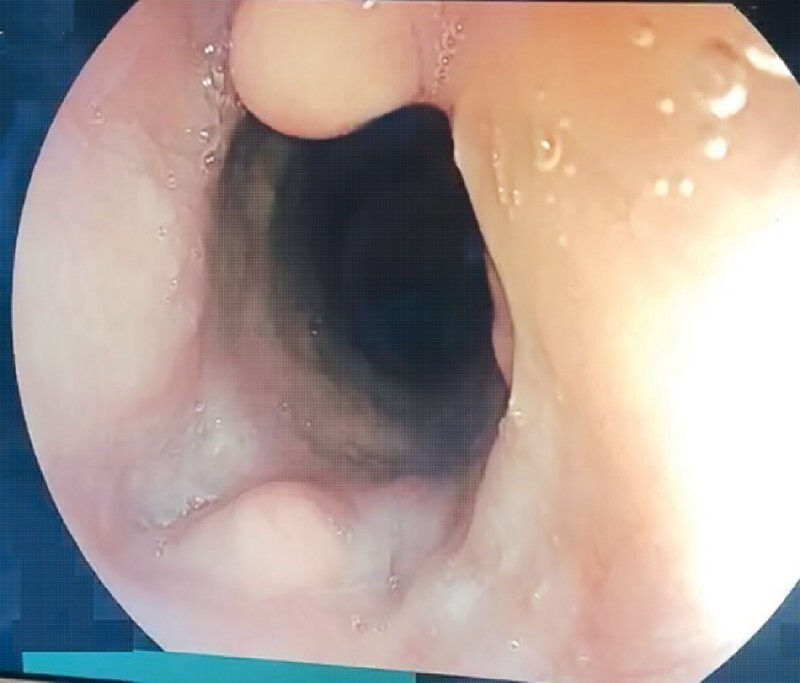
Complete disruption of esophageal mucosal bridge with healed mucosa was noted on follow-up endoscopy.

This case highlights endoscopic management of EMB, a rare cause of dysphagia. EMB management using a scissor-type knife is safe and provides durable clinical improvement.

Endoscopy_UCTN_Code_TTT_1AO_2AG

## Notes

Endoscopy E-Videos

https://eref.thieme.de/e-videos
*E-Videos* is an online section of the journal *Endoscopy* , reporting on interesting cases and new techniques in gastroenterological endoscopy. All papers include a high-quality video and are published with a Creative Commons CC-BY license. Endoscopy E-Videos qualify for HINARI discounts and waivers and eligibility is automatically checked during the submission process. We grant 100% waivers to articles whose corresponding authors are based in Group A countries and 50% waivers to those who are based in Group B countries as classified by Research4Life (see: https://www.research4life.org/access/eligibility/ ). This section has its own submission website at https://mc.manuscriptcentral.com/e-videos
